# Galectins as Molecular Targets for Therapeutic Intervention

**DOI:** 10.3390/ijms19030905

**Published:** 2018-03-19

**Authors:** Ruud P. M. Dings, Michelle C. Miller, Robert J. Griffin, Kevin H. Mayo

**Affiliations:** 1 Department of Radiation Oncology, University of Arkansas for Medical Sciences, Little Rock, AR 72205, USA; rpmdings@uams.edu (R.P.M.D.); rjgriffin@usma.edu (R.J.G.); 2 Department of Biochemistry, Molecular Biology & Biophysics, University of Minnesota, Minneapolis, MN 55455, USA; mill0935@umn.edu

**Keywords:** galectins, carbohydrates, apoptosis, cell adhesion, protein structure, cancer, galectin-targeted therapeutics

## Abstract

Galectins are a family of small, highly conserved, molecular effectors that mediate various biological processes, including chemotaxis and angiogenesis, and that function by interacting with various cell surface glycoconjugates, usually targeting β-galactoside epitopes. Because of their significant involvement in various biological functions and pathologies, galectins have become a focus of therapeutic discovery for clinical intervention against cancer, among other pathological disorders. In this review, we focus on understanding galectin structure-function relationships, their mechanisms of action on the molecular level, and targeting them for therapeutic intervention against cancer.

## 1. Galectins from a Structural Perspective

Galectin-1 (Gal-1) has been the most studied and well-characterized galectin [1], since it was the first galectin discovered by its display of hemagglutinating activity [2]. Currently at least 14 mammalian galectins have been reported, and many more are found in different organisms, e.g., vertebrates, inter-vertebrates, and protists [3,4,5]. Early on following the discovery of galectins, it was proposed that they be divided into three groups [6,7]: prototype (galectin-1, -2,-5, -7, -10, -11, -13, -14), chimera (galectin-3), and tandem repeat (galectin-4, -6, -8, -9, -12). Prototype galectins consist of a single core domain, usually referred to as the carbohydrate recognition domain (CRD). Galectin-3 (Gal-3) is the only chimera galectin known, and it has a CRD and a collagen-like N-terminal tail with different properties. Tandem repeat galectins have two homologous, yet distinct, CRDs that are connected to each other via linker polypeptide chains.

**Figure 1:**
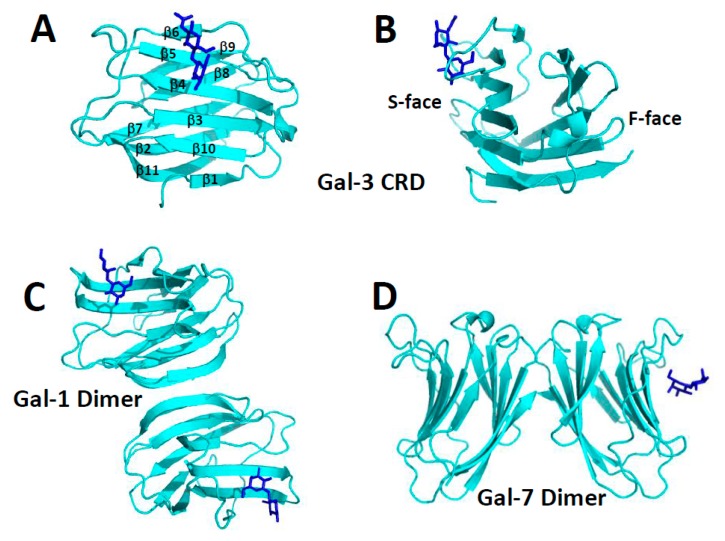
Galectin oligomer states. (**A**,**B**) Gal-3 carbohydrate recognition domain (CRD) (Protein Data Bank (PDB) access code 1A3K) as a monomer illustrating the β-sandwich fold common to all galectins. The 11 β-strands found within the CRD β-sandwich are labeled β1 to β11 in **A**, and the S- and F-faces of the CRD are identified in **B**. (**C**,**D**) Two prototype galectin dimers are shown. The CRD of the Gal-1 “terminal” dimer (PDB access code 1GZW) is shown in **C**, and the “symmetric sandwich” dimer of human Gal-7 (PDB access code 1BKZ) is shown in **D**. The carbohydrate binding sites in all structures are indicated by the lactose molecules shown in blue with a ball-and-stick structure.

All three types of galectins (prototype, chimera, and tandem repeat) have a well-defined CRD with a highly conserved amino acid sequence and β-sandwich structure [8]. The CRD β-sandwich structure is composed of eleven β-strands (β1 to β11) running in antiparallel fashion, with six of them (β1, β10, β3, β4, β5, β6) defining the sugar binding face (S-face) of the CRD and the remaining five (β11, β2, β7, β8, β9) defining the opposing F-face, as illustrated with the Gal-3 CRD in Figure 1A,B. High resolution structures of the CRDs of many galectins (usually bound to lactose or *N*-acetyl-lactosamine) have been reported: e.g., human galectin-1 (Gal-1, [9]), galectin-2 (Gal-2, [10]), CRD of Gal-3 [11,12], C-terminal CRD of galectin-4 (Gal-4, [13]), galectin-7 (Gal-7, [14]), galectin-10 (Gal-10, [15,16,17]), bovine Gal-1 [18,19], mouse galectin-9 (Gal-9, [20]), toad ovary galectin [21,22], chicken galectin-16 (Gal-16, [23], galectins from conger eel: congerin I [24] and congerin II [25,26], fungal galectins from *Coprinopsis cinerea* [27] and *Agrocybe cylindracea* [28]. 

The largest group of galectins (e.g., Gal-1, -2, -5, -7, -10, -13) belong to the prototype class and are generally known to self-associate, mostly as dimers [8]. Moreover, irrespective of their conserved monomer folds, galectins can form different types of dimers. The “terminal” dimer typified by Gal-1 is formed by hydrophobic interactions between N- and C-terminal residues of two subunits related by a 2-fold rotation axis perpendicular to the plane of the two β-sheets (Figure 1C). There are also “symmetric” and “non-symmetric” sandwich dimers. The former (e.g., Gal-7, Figure 1D) is stabilized by electrostatic interactions among charged residues on the F-faces of two monomers, and its inter-subunit contact surface is reduced compared to that in the non-symmetric dimer. The “non-symmetric” dimer (e.g., Gal-2) interface involving β-strands β1 and β6 from each subunit, is also formed primarily by electrostatic interactions at the inter-subunit interface of two monomers. 

The Gal-1 dimer is the most thermodynamically stable of all galectins (dimer dissociation constant, *K_d_* ~ 2–7 × 10^−6^ M) [29,30]. Dimers of other prototype galectins are generally less thermodynamically stable. For example, Gal-5 and Gal-7, even at intermediate concentrations, behave as monomers [31,32], even though Gal-5 can induce cell agglutination, suggesting the presence of self-association. In the crystal, Gal-7 appears to be a dimer [14], whereas in solution others have reported it to be either a monomer [4,14,33] or dimer [14,34,35]. In addition, Gal-10 can form Charcot-Leyden crystals in tissues and during secretion [16]. Whereas most galectins dimerize via non-covalent interactions, Gal-13 dimer subunits are covalently linked via disulfide bonds which when reduced abrogate cell agglutination function. In many of these instances, the solution environment can influence the degree of self-association.

Because CRD structures are highly conserved, formation and thermodynamic stability of a prototype galectin dimer result from the composition of amino acid residues at the inter-subunit interface [8,36]. When the free energy of interaction of one type of dimer is greater than that of another, the greater one will of course dominate in solution. Thus, the type of dimer formed is likely to be functionally important in terms of defining how different galectins bind to glyco-conjugates on the cell surface. Moreover, based on this same thermodynamic argument, different galectins have recently been reported to form heterodimers with potential biological consequences [37].

The only chimera Gal-3 (30 kDa) has a C-terminal CRD linked to a lengthy, collagen-like, dynamic and structurally aperiodic N-terminal tail (NT, 113 amino acid residues in human Gal-3) that is comprised of numerous proline and glycine residues (27 each in human Gal-3) usually found in “PGAY” tetrapeptide repeats [8]. Ippel et al. (2016) [38] found that the Gal-3 NT binds transiently to the F-face of the CRD with these tetrapeptide repeats being crucial to those interactions. Even though Gal-3 oligomerization has been proposed [39], its oligomeric state remain unclear. Gal-3 has been reported to be a monomer [40], a dimer [41,42], and a higher order oligomer state [43,44] that is possibly formed by chemically cross-linking [45] through the action of transglutaminase [46]. When bound to some synthetic carbohydrates, Gal-3 has been reported to precipitate from solution as a pentamer by interactions among its N-terminal non-lectin domain, presumably to enhance cross-linking of cell surface oligosaccharides [47]. Nevertheless, this model generally lacks experimental validation. 

Tandem-repeat type Gal-4, -6, -8, -9 are comprised of two CRDs connected by a variable length linker peptide. Even though this class of galectins is usually reported to be monomeric, a few studies have reported that tandem-repeat Gal-9 self-associates as dimers (mouse Gal-9 [20]) or multimers (human Gal-9 [48]). Nevertheless, because tandem-type galectins have two CRDs, they effectively mimic the function of prototype galectin dimers in terms of cross-linking cell surface glycoconjugates. In any event, this suggests some level of biological control and/or evolutionary link, in that tandem-repeat type galectins cannot dissociate into single CRD monomers. The presence of two CRDs appears necessary to mediate full cross-linking function in terms of mediating cell adhesion and migration.

## 2. Carbohydrate Binding

At least extracellularly, galectins generally function by binding to the carbohydrate portion of glycoconjugates on the cell surface [8]. The galectin CRD carbohydrate binding site comprises highly conserved amino acids within the six-stranded β-sheet on the S-face (Figure 1A,B) [49]. Even though most studies have been performed with small β-galactosides such as lactose, glycoconjugates in situ on the cell surface to which galectins bind, are more complicated, which may also play a role in differentiating galectin function. Moreover, even though it appears that galectins generally recognize β-galactosides as the binding epitope, Gal-1 has been reported also to interact with some α-galactosides, albeit more weakly than to the β-galactoside lactose [50].

Lactose is the minimal carbohydrate ligand necessary for binding to galectins, and most structures of galectins are reported with lactose bound. Most CRD residues required for optimal interactions with carbohydrate ligands are conserved arginines and histidines, along with a single conserved tryptophan. Lactose is effectively “grabbed” by the peptide loop above the lactose molecule and the relatively large and flat tryptophan side chain at the bottom of the disaccharide. NMR structural studies indicate that this loop is relatively flexible when the disaccharide is absent and is more firmly positioned when the disaccharide is bound with other CRD residues becoming more mobile, thus contributing to conformational entropy and a more negative free energy of binding [36]. 

Lactose binding affinity to galectins usually falls in the micromolar to millimolar range (e.g., 64 × 10^–6^ M for Gal-1 [51] to 2 × 10^–3^ M for nematode galectin LEC-6 CRDs [52]. For Gal-3, the reported range is quite broad, e.g., 26 × 10^–6^ M [52], 1 × 10^–3^ M [53], and 0.6 × 10^–3^ M [45]. For Gal-2, this value was reported as 85 × 10^–6^ M [27], and for galectin-4, it is 0.9 × 10^–3^ M [54]. Overall, these *K_d_* values indicate relatively weak binding of lactose, the minimal unit necessary for carbohydrate recognition by galectins. Binding affinity can be increased by modifying the disaccharide. *N*-acetyllactosamine binds e.g., about 5-fold better to Gal-3 (*K_d_* of 0.2 × 10^–3^ M) and binds even more strongly to larger oligosaccharides [53,55], such as β(1,3)-linked polyNAc-lactosamino-glycan as found in the extracellular matrix and many cell surface glycoconjugates. 

There are specific structural features in oligosaccharides that promote stronger binding to the CRD, and binding affinities can usually be explained by some carbohydrate recognition features [52]. The basic unit recognized by all galectins is Gal-β(1-4)-GlcNAc, although *K_d_* values vary greatly with a particular galectin. Moreover, isomers of this disaccharide can modify binding affinities, e.g., Gal(1-3)GlcNAc. Structurally, configuration of the 3-OH group is essential for carbohydrate recognition, and substitution of 4-OH and 6-OH groups on the galactose ring usually attenuates binding. These three hydroxy groups in lactose (or *N*-acetyl-lactosamine) form hydrogen bonds with side chains of hydrophilic residues from galectins [25]. The galactose ring in particular forms several H-bonds between its oxygen atoms O4, O5 and O6, and H44, E71, and N61 of Gal-1. The O3 of the glucose ring forms H-bonds with residues R48 and E71. H52 and W68 make van der Waals interactions with both the glucose and galactose rings, respectively, in lactose. Because of this, galectins generally do not bind to terminal, non-reducing end mannoside or glucoside residues, and substitution at the 3(4)-OH of the penultimate saccharide can abolish binding.

The non-polar side of the galactose ring (i.e., H1, H3, and H5) interacts with the highly conserved tryptophan residue present with the carbohydrate binding sites in all galectins. In glucose, the C4-OH group is equatorial, which attenuates hydrophobic interactions with this tryptophan [56]. Due to these crucial interactions with the galactose ring, the remainder of the polysaccharide in longer carbohydrates is oriented away from the protein surface and out into solution [18,27]. Furthermore, changes at the 3′ position of the β-galactoside *N*-acetyllactosamine with sialic acid increases binding affinity compared to *N*-acetyl-lactosamine (LacNAc), and addition of the α(1-2)-fucoside increases it further. When immobilized, linear B2 trisaccharide and Galili pentasaccharide are some of the best ligands with *K_d_* values ~ 1 × 10^–6^ M, along with more complex *N*-acetyllactosamine-based oligosaccharides (e.g., -3Galβ1-4GlcNAcβ1-)_n_ sequences), complex-type biantennary *N*-glycans, and modified chitin-derived glycans that display similar *K_d_* values ~ 2 to 4 × 10^−6^ M [57]. However, when free in solution, these glycans bind more weakly, suggesting that the binding epitope on surface-bound glycans is conformed for more favorable galectin interactions. In some galectins (e.g., Gal-1,-3 and -9), oligosaccharide branching can also enhance binding affinity [52], whereas in others (e.g., Gal-8) is can result in decreased affinity. Thus, branching is likely one other way in which galectins can modulate their activities.

Gal-1 and Gal-3 have also been reported to interact with relatively large polysaccharides. A 120 kDa rhamnogalactouronan was found to bind Gal-1 at the CRD S-face with the actual carbohydrate binding site being more extensive than for simple disaccharides, a finding that has implications for interactions between galectins and glycans on the cell surface [58]. Miller et al. [59] also reported that a ~60 kDa α-galactomannan binds Gal-1 at the F-face of the CRD, a site different from the S-face canonical carbohydrate binding site. Moreover, the binding epitope on this α-galactomannan most likely involves a disaccharide unit comprised of α-(1-6)-galactose-linked residues on the mannan backbone that is flanked by “naked” mannan regions [60]. More recently, Miller et al. [61] reported that this α-galactomannan interacts in a similar fashion with the Gal-3 CRD. Aside from this novel polysaccharide binding site on the CRD F-face of Gal-1 and -3, Miller et al. [62] found that another rhamnogalacturonan polysaccharide (RG-I-4, ~60 kDa) could bind relatively strongly to the N-terminal sequence of the Gal-3 NT, with strong binding occurring kinetically slowly that is most likely associated with proline cis trans isomerization.

## 3. Galectin Function

**Figure 2:**
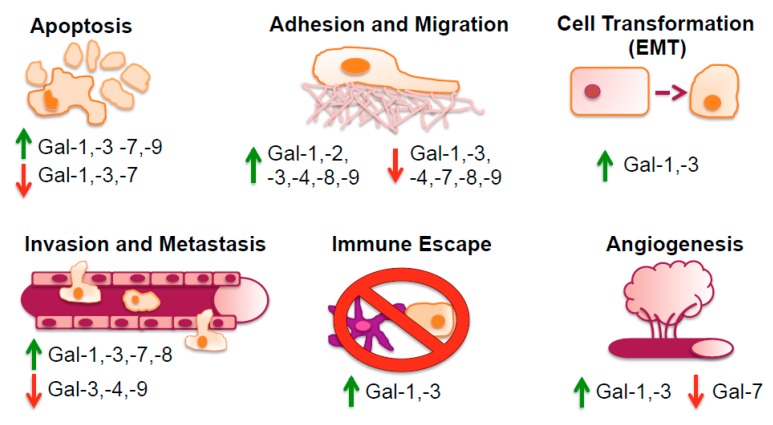
Galectins are involved in multiple processes of cancer initiation and development. A diversity of galectins is associated with key aspects of carcinogenesis, including apoptosis, adhesion and migration, cell transformation (EMT), invasion and metastasis, immune escape, and angiogenesis. Their tentative roles can be pro- and/or anti-tumorigenic, as indicated by green arrows up and red arrows down, respectively.

Galectin expression, which varies considerably from cell type to cell type, depends upon the activation state of a certain cell type. All cells appear to express at least one galectin, and each galectin tends to be expressed at high concentration in a few, but different cell types [44]. Galectins can be translocated to the nucleus or to other sub-cellular sites after being synthesized on cytosolic ribosomes. Galectins have several features in common with cytosolic proteins, such as being deficient in a secretion signal peptide or typical transmembrane segments, and they can have acetylated *N*-termini. This diversity in their occurrence is also reflected in the multimodal biological roles they exhibit in controlling cell-cell and cell-matrix interactions, adhesion, proliferation, apoptosis, pre-mRNA splicing, immunity, and inflammation [63], as illustrated in Figure 2. The underlying principle of all these functions is most often, but not always, carbohydrate recognition.

The extracellular mechanism of action of galectins generally starts with their binding to saccharides associated with cell surface glycol-conjugates [64]. However, the overall function of any given galectin can vary considerably. For example, Gal-1 can induce T-cell apoptosis, whereas Gal-3 can suppress apoptosis and increase T-cell proliferation [65,66]. Therefore, the activity of any galectin can be multi-faceted, and galectin self-association and interactions with cell surface glycans, as well as interactions with other biomolecules in situ, both extracellularly and intracellularly, can have significant impact on galectin function. For example, Gal-1-induced mitogenicity of human fibroblasts is attenuated as its concentration is increased (i.e., greater dimer population) [67], suggesting that monomers mediate mitogenic activity. On the other hand, the effect of Gal-1 on the growth of fibroblasts and human epithelial (HEP) 2 carcinoma cells is enhanced at high concentrations where the dimer population is greater [67]. 

Galectins bind numerous glycoconjugates on the surfaces of different cells, an event that impacts on their function. For example, Gal-1 interacts with various glycoconjugate ligands of the extracellular matrix (e.g., laminin, fibronectin, integrins, and ganglioside GM1), as well as those on endothelial cells (e.g., integrins, ROBO4, CD36, and CD13) [68] and on T lymphocytes (e.g., CD7, CD43, and CD45) where it promotes apoptosis [69]. Gal-2 can induce exposure of cell membrane surface phosphatidylserine in activated neutrophils, but not in activated T-cells [70], and has been associated with binding to lymphotoxin-α and myocardial infarction [71]. Gal-7 is associated with p53-induced apoptosis in keratinocytes [72], as well as in colon carcinoma [73]. Gal-8 is the most abundant galectin in tumor cells of different origin [74], and is closely related to prostate carcinoma tumor antigen-1 (PCTA-1) [75]. Gal-8 binds to gangolioside GM_3_ (sialosyllactoseceramide) that associates with CD9 and CD82 to promote an anti-metastatic effect [76,77]. 

Gal-3 interacts with glycoconjugates in the extracellular matrix, such as laminin, fibronectin, vitronectin, elastin, neural cell adhesion molecule (N-CAM), lysosomal-associated membrane protein (LAMP) 1 and 2, and integrin α_3_β_1_. Like Gal-1, Gal-3 can also bind to CD43 and CD45 on leukocytes, as well as to CD66, immunoglobulin E (IgE), IgE receptor, and Mac-2 binding protein. Besides its constitutive expression, Gal-3 can be induced by inflammatory mediators [78], such as chemokine CXCL8 [79]. Functionally, Gal-3 appears to be the most promiscuous galectin, exhibiting diverse biological activities from cell adhesion, apoptosis, immune regulation, to regulation of gene transcription.

The quaternary structure of prototype galectins can also lead to functional divergence [80]. For example, Nieminen et al. [81] reported that Gal-3 oligomerization mediates cell activation/repression and cell adhesion via three different modes of action: receptor clustering, lattice formation, and cell-cell interactions. In addition, for prototype galectins, formation of dimer type (terminal, symmetric, and non-symmetric) can be functionally important, since this may help differentiate how different galectins bind differently to complex glycans intra- or extracellularly on the surface of cells. These natural glycans are far more complex than simple disaccharides such as lactose that have been used to study galectin carbohydrate binding and function. Lactose binding to the Gal-1 and -7 dimers has also been shown to modify functional binding at one carbohydrate binding site can allosterically influence lactose binding to the other, either with positive or negative cooperativity, thus providing another angle for galectin functional divergence [36,82]. 

The functional importance of the Gal-1 oligomer state is also evidenced by a naturally-occurring form of Gal-1 (Gal-1β) that lacks the first 6 N-terminal residues [83] and remains essentially monomeric [76]. The Gal-1β monomer promotes axonal regeneration, but not Jurkat cell death, unlike dimer-forming Gal-1, which promotes both [76]. 

Galectins can also function intracellularly. e.g., Gal-3 can be found in the nucleus and cytoplasm as a multifunctional oncogenic protein that can associate with Ras [84], and other cytosolic moieties such as Bcl-2 to help regulate cell growth and apoptosis, an interaction that can be abrogated by carbohydrate binding [65]. Gal-1 can also interact with H-Ras to enhance its association with the intracellular membrane to modulate H-Ras-GTP loading [85], an activity of Ras that is dependent intracellular membrane anchorage via hydrophobic interactions, possibly with the farnesyl group covalently attached to Ras [84,86,87,88]. Gal-3 also interacts with thyroid-specific transcription factor TTF-1, suggesting a role for this lectin in controlling proliferation and tumor progression in thyroid cancer [89]. Gal-3 also interacts with synexin (annexin VII, a Ca^2+^ and phospholipid-binding protein) that mediates Gal-3 translocation/trafficking to the perinuclear mitochondrial membrane, where it regulates mitochondrial integrity and cytochrome *c* release critical for apoptosis regulation [90].

**Figure 3:**
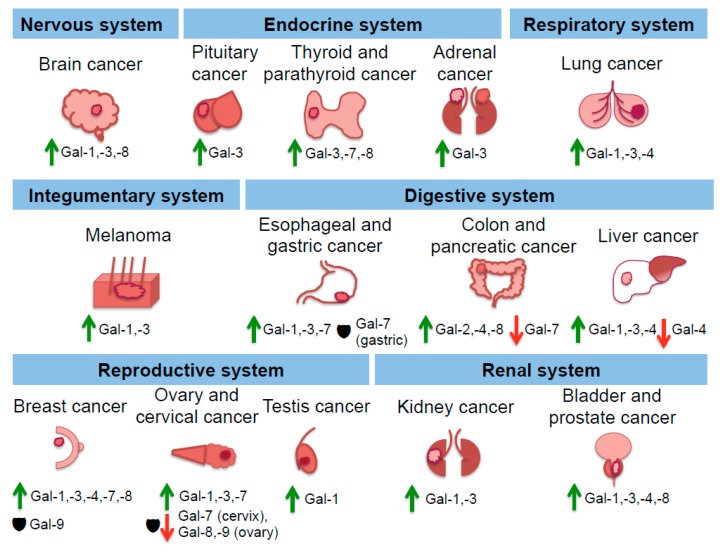
Involvement of galectins in organ-specific carcinogenesis within different physiological systems. Various elevated galectin expression is associated with tumor progression (green arrow up). In some instances, low galectin expression is correlated with the generation of neoplastic tissue (red arrow down). Additionally, galectins can have potential protective roles (black shield) during carcinogenesis.

In regards specifically to cancer, multiple investigations have uncovered the various roles and mechanisms of action of galectins in tumor cell invasiveness and dissemination. The increased and decreased levels of some galectins in different cancers are illustrated in Figure 3. Based on clinical data the presence of e.g., Gal-1 has been correlated with increased rates of metastasis and poor patient survival outcome [91]. Gal-1 induces epithelial-mesenchymal transition (EMT) in multiple cancer types by various signaling pathways [91,92,93,94,95,96,97]. Tumor cells undergoing EMT differentiate into a mesenchymal state, often indicated by molecular markers such as vimentin, desmin and α-smooth muscle actin (α-SMA). Functionally, this mesenchymal state increases the cellular ability and probability for tumor cell migration and invasion via metastasis [91]. E.g., Bacigalupo et al. [92] noted that in hepatocellular carcinoma (HCC) cell line HepG2 Gal-1-associated EMT was mediated through β-catenin nuclear translocation, TCF4/LEF1 transcription activity and increased cyclin D1 and *c-Myc* gene expression, implying the involvement of the Wnt pathway. Zhang et al. elucidated that artificially inducing Gal-1 expression triggered EMT through the α_v_β_3_-integrin/FAK/PI3K/AKT signaling pathway [91].

Conversely in kidney cancer, elevated levels of Gal-1 induce nuclear factor (NF)-κB signaling thereby inducing chemokine CXCR4 expression [93]. This has also been demonstrated in glioblastoma in which EMT is triggered via the stromal cell-derived factor-1 (SDF-1)/CXCR4 axis [94]. In gastric cancer, however, overexpression of Gal-1 enhances the ability of gastric cancer cells to invade and metastasize via EMT through the non-canonical hedgehog pathway, increasing the transcription of glioma-associated oncogene-1 by a smoothened independent manner [95]. Aside from the effect of Gal-1 on the gastric tumor cells, the stroma is also affected by Gal-1. In this regard, conditioned media from gastric cancer cells induces expression of Gal-1 and the EMT marker α-SMA in normal fibroblasts, thus causing normal fibroblast transformation into mesenchymal cancer-associated fibroblasts via a transforming growth factor-β (TGF-β) dependent mechanism and the progression of gastric tumors [96]. Thus, it appears that Gal-1 can induce EMT through multiple pathways on both the tumor parenchyma and stroma. In clinical samples of pancreatic ductal adenocarcinoma, immunohistochemical analysis has revealed a positive correlation of Gal-1 with the expression of EMT markers [97]. By means of knockdown and overexpression in pancreatic cancer cell line PANC-1 and co-cultures with activated pancreatic stellate cells, EMT was induced by the NF-κB pathway, stimulating malignant behavior of pancreatic ductal adenocarcinoma [97].

Other galectins, such as Gal-3, have also been implicated in the EMT [98,99]. The Gal-3 EMT-associated phenotype was observed in patients with stage II colon cancer. Here, elevated tumoral expression of Gal-3 was positively correlated with vimentin expression and negatively correlated with E-cadherin expression when compared to the surrounding normal tissue [99]. Univariate analysis revealed that this EMT phenotype (i.e., elevated Gal-3 and vimentin expression) are predictors of tumor recurrence and survival [99]. Similarly, in patients with oral tongue squamous cell carcinoma (OTSCC), Gal-3 was found to be over-expressed in OTSCC compared to normal adjacent tissue, which was correlated strongly with the pathological stage, grade, and lymph node invasion in a Wnt/β-catenin dependent manner [100]. This has been similarly reported for Gal-1 in hepato carcinoma cells (HCC) [92]. At least in fibrosis, the EMT was counteracted by inhibition of Gal-3 by using modified citrus pectin (MCP), as shown by the reduction of mesenchymal molecules, fibronectin, smooth muscle actin and β-catenin, as well as hypertension and fibrosis [98]. Whether MCP can reverse Gal-3-induced EMT in cancer has yet to be demonstrated.

Gal-8 is the most abundant galectin in tumor cells of different origin [74], and is closely related to prostate carcinoma tumor antigen-1 (PCTA-1) [75]. Gal-3 is also reported to be a substrate for prostate-specific antigen (PSA) [101], and some of its glycoprotein ligands have been associated with prostasomes [102,103,104]. These findings suggest that Gal-8 and Gal-3 antagonists may be effective against reproductive and prostate cancer. On the other hand, low expression levels of Gal-3 have been associated with EMT and lymphovascular invasion and overall survival in lymph node positive breast cancer patients treated with doxorubicin [105]. In vitro assays using Gal-3 knockdown breast cancer stem cells have been shown to enhance tumorigenicity, which was confirmed in orthotopic mouse models [105]. Recently, Gal-8 has also been associated with EMT [106]. Non-tumurogenic Madin-Darby canine kidney (MDCK) cells acquired oncogenic potential after Gal-8 overexpression, displaying hallmarks of EMT alongside: downregulation of E-cadherin and upregulation of vimentin, fibronectin, β-catenin, and transcription factor Snail. Intriguingly, this EMT phenotype was considered partial and reversible because confluency was able to revert the EMT phenotype [106]. 

Overall, clinical data and experimental molecular studies have shown that galectins display various roles and mechanisms of actions within the EMT, ultimately causing increased cancer invasiveness and dissemination. Therefore, one or more of these galectins are potential molecular targets for therapeutic development.

## 4. Galectin Antagonists

For some time, many labs have sought to identify, discover, or design various galectin antagonists. Given its promiscuous nature, Gal-3 has been perhaps the most focused molecular target, and Gal-1 is a close second, because it is not usually involved in normal physiological processes such as wound healing [107] and yet is highly expressed in human tumors [108]. Nevertheless, antagonists against other galectins have also been identified. 

Most reported galectin antagonists are based on the disaccharides lactose or *N*-acetyllactosamine, with the design aim of targeting a single galectin. Nevertheless, specificity and in vivo exposure remain as obstacles to developing highly effective galectin antagonists with therapeutic value in the anti-inflammatory [109] and anti-cancer arenas [110]. Some examples of galectin inhibitors are 3-(1,2,3-triazol-1-yl)-1-thio-galactosides (best *K_d_* ~ 107 × 10^−6^ M) [111], *O*-galactosyl aldoximes (best *K_d_* ~ 180 × 10^−6^ M) [112], and phenyl thio-β-d-galactopyranosides (best *K_d_* ~ 140 × 10^−6^ M) [113]. Specificity with all of them was essentially absent. E.g., the phenyl thio-β-d-galacto-pyranosides interacted with all screened galectins (i.e., Gal-1, -3, -7, -8, and -9), with the best one binding most strongly to Gal-7. Thioureido *N*-acetyllactosamine derivatives were screened as inhibitors of Gal-7 and -9, with the best ones exhibiting *K_d_* values of 23 × 10^−6^ M and 47 × 10^−6^ M, respectively [114]. 

Increased specificity for Gal-3 was observed upon addition of an aromatic (arene) group in lactose-based compounds having an aromatic 4-methoxy-2,3,5,6-tetrafluoro-benzamido moiety [12]. In the co-crystal structure with the Gal-3 CRD, the arene group improved affinity by stacking against Arg144 within the carbohydrate binding site of the CRD [12]. Further structure-based design of this compound class produced an analog with even greater affinity for Gal-3 (*K_d_* ~ 0.32 × 10^−6^ M), and double arene thiodigalactoside bis-benzamido analogs improved affinity and specificity for Gal-3 further, because the two arene groups were observed in crystal structures to interact with two arginine residues (Arg144 and Arg186) [115]. In this case, the best analog had a *K_d_* of 33 × 10^−9^ M [115]. The theme of adding a hydrophobic group to a lactose-based compound to improve Gal-3 inhibitor affinity and specificity was also used in the attachment of linear alkyl chains of varying length to the anomeric carbon of the glucose or *N*-acetyl glucose ring [116]. Although this class of compounds did increase affinity (best *K_d_* values of 11 × 10^−6^ M to 73 × 10^−6^ M) over lactose [116], they were not as impressive as some compounds from the arene class [12,115]. 

Binding multivalency has also been exploited in designing galectin antagonists. By functionalizing unnatural amino acids (phenyl-bis-alanine and phenyl-tris-alanine) with 2-azidoethyl β-d-galactopyranosyl-(1-4)-β-d-glucopyranoside, a relatively effective compound targeting Gal-1 in particular has been found with a *K_d_* of 3.2 × 10^−6^ M, about one order of magnitude higher affinity than for other galectins tested [117]. The multivalent design approach has been used in a number of other instances, namely with a trivalent “lactose” analog against Gal-4 (*K_d_* of 22 × 10^−6^ M) [117], a bilactosylated steroid-based compound against Gal-1 [118], and lactulose amine compounds (i.e., polymethylene-spaced dilactoseamine derivatives) that show some selective effects linked to tumor cell apoptosis, cell aggregation, and endothelial cell morphogenesis [119].

The list of galectin antagonists is increased with the design of glycomimetics using high resolution structures of Gal-1 and -3 and computational approaches (e.g., Quantitative Structure-Activity Relationship (QSAR) models) to correlate molecular properties and binding affinities and to conclude that selective and potent inhibitors could be engineered by modifying the carbohydrate C-3′ and O-3 positions [120,121]. This approach led to the synthesis of aryl *O*- and *S*-galactosides and lactosides, as well as triazoles and isoxazoles, with the best compounds having *K_d_* values in the 20 to 40 × 10^−6^ M range [117,121,122]. 

Many of these carbohydrate-based antagonists have provided evidence that subtle differences in saccharide structure can be used to fine tune binding affinity and specificity and may be potentially useful to block tumor growth. Nevertheless, galectin binding specificity and relatively low binding affinity remain problematic, in particular when developing a therapeutic with acceptable in vivo exposure. In this regard, clinical use has most often been limited to use in pathological disorders where topical administration can be done. For example, the Leffler lab has reported a small molecule and galactose-coumarin-based, Gal-3 inhibitor that reduces corneal neovascularization and pulmonary fibrosis in animal models [123,124]. Some other saccharide-based agents have potential to be developed as therapeutics for clinical use. Recently some reported thiodigalactoside, fluorine-amide and phenyl-arginine derivatives were reported with low nanomolar binding affinity and relatively high selectivity for Gal-3 [125,126]. 

A complex polysaccharide (a rhamnogalactouronan, termed GR-MD-02) has emerged as a relatively potent Gal-3 antagonist with considerable promise in preclinical animal models and clinical trials against non-alcoholic steatohepatitis (NASH) and toxic cirrhosis, demonstrating action at multiple pathophysiological processes [127,128,129,130]. GR-MD-02 also has recently been found to display good efficacy against cancer in pre-clinical cancer models when used in combination with immunotherapy agents. Moreover, an investigator-initiated phase 1b clinical trial with GR-MD-02 in combination with Merck’s therapeutic drug KEYTRUDA (pembrolizumab) was shown to be effective against advanced melanoma with 5 of 8 responders (2 complete responders (CR) and 3 partial responders (PR)) in advanced melanoma (Galectin Therapeutics Inc., Atlanta, GA, USA). In addition, GR-MD-02 in combination with the anti-OX40 immunotherapy agonist was shown to improve survival and reduce lung metastases in the 4T1 breast cancer model, as well as improving survival in the mouse sarcoma cell (MCA-205) model in a CD8 T cell-dependent fashion. Another polysaccharide, an α-galactomannan, that targets and binds to the F-face of Gal-1 and -3 could be another potentially good cancer therapeutic [58,60]. 

Several galectin-targeting peptides have also been reported. For example, two peptides (G3-A9 and G3-C12, with amino acid sequences PQNSKIPGPTFLDPH and ANTPCGPYTHDCPVKR, respectively) were shown to bind relatively specifically to Gal-3 (*K_d_* of 80 × 10^−9^ M) and recognize cell surface Gal-3 on carcinoma cells and monocytes, block the interaction between Gal-3 and TFAg (Thomsen-Friedenreich glycoantigen), and inhibit adhesion of human breast carcinoma cells to endothelial cells [131]. Another designed peptide anginex (33 amino acid residues) that targets Gal-1 (*K_d_* ~ 90 × 10^−9^ M) [108] displays multimodal activities in terms of inhibiting tumor endothelial cell (EC) adhesion, migration and proliferation [132,133] and promoting leukocyte infiltration into tumors [134,135] leading to tumor growth inhibition in mouse models [136,137,138]. In this regard, anginex interferes with Gal-1 function by preventing tumor angiogenesis [108], abrogating tumor escape from immunity via blockade of Gal-1-induced apoptosis in activated T lymphocytes [139], and preventing metastasis via inhibition of Gal-1-facilitated tumor cell-EC interactions [140]. Anginex is unique in that rather than targeting the carbohydrate binding site on the CRD (S-face), it binds to a mostly hydrophobic patch on the CRD F-face [141]. A structure-based approach [142,143,144] was then used to design both a partial peptide mimetic [145] and a fully non-peptide, calixarene-based protein surface topomimetic of anginex called PTX008 [141,146]. Both agents are antagonists of Gal-1 function, with improved activity over anginex [144]. PTX008 has been used in the clinic in a Phase I trial against cancer and has shown some efficacy at inhibiting tumor growth. 

## 5. Galectin Antagonists in Combination Therapy 

Whereas galectin antagonists may take only a limited share of therapeutic space as stand-alone agents, their real forte is likely to be in combination therapy.

### 5.1. Improving Chemo- and Immunotherapy 

Treatment modalities targeting tumor stroma have been shown to transiently normalize tumor vasculature, which can alleviate hypoxia, increase drug and anti-tumor immune cell delivery, and consequently improve clinical outcome [141,147,148,149,150,151]. Initially vessel normalization was thought to be achieved only by interfering with growth factor signaling, e.g., blocking vascular endothelial cell growth factor receptor 2 (VEGFR2) or basic growth factor signaling, causing destruction of ‘‘immature’’ vessels and thus improving the overall physiologic state of the tumor over a relatively short period of time [152,153,154]. Vessel normalization is defined by an increase in pericyte coverage of tumor vasculature and functionally by modifying tumor perfusion and interstitial pressure, resulting in increased tumor oxygenation. Anti-angiogenesis therapy can overcome endothelial cell anergy and promote leukocyte-endothelium interactions and infiltration in tumors [134].

In addition to growth factor interference, the inhibition of Gal-1 can be a potent approach to vessel normalization. The Gal-1 inhibitor anginex has been shown to normalize tumor vasculature and consequently elevates tumor oxygenation in multiple tumor models [137,155,156]. More recently, this finding was corroborated in mice bearing Kaposi’s sarcomas by using a rabbit anti-Gal-1 IgG antibody (F8.G7), which was also able to transiently normalize tumor vasculature as evidenced by vasculature remodeling: increased pericyte coverage of vessels causing an improved tumor physiology indicated by reduced tumor hypoxia and improved T-cell infiltrate [157]. Anginex has also been conjugated to the cytotoxic acylfulvene, 6-hydroxylpropylacyl-fulvene to inhibit tumor growth more effectively [158]. In addition, the non-peptidic calixarene-based mimetic of anginex (PTX008), which specifically binds Gal-1 in an allosteric fashion, has been demonstrated to normalize tumor vasculature, thus promoting improved tumor oxygenation [141,146,159,160]. In addition, anginex and PTX008 in combination with the chemotherapeutic irofulven have been shown to lead to ovarian tumor growth regression [151]. The use of these anti-angiostatic agents has also been shown to enhance T-cell mediated anti-tumor response when used as an adjuvant to immunotherapy [161]. 

Thus, peptide-based, antibody-based, and small molecule-based Gal-1 inhibitors can transiently normalize tumor vasculature to increase the sensitivity of tumors to chemo- and immunotherapy and have the potential to significantly enhance clinical success.

### 5.2. Drug Resistance

One problem in the clinic with galectin-targeted therapeutics (as well as with any therapeutic agent) against cancer is the issue of drug resistance. This occurs for various reasons, e.g., tumor generated multiple isoforms via alternative splicing that may result in inhibitor-resistant galectins [162]. Overexpression of Gal-1 has been positively correlated with poor survival outcome, as well as drug and radiation resistance [91,163,164,165]. Additionally, multidrug resistant (MDR) breast cancer cell lines showed improved sensitivity to paclitaxel and adriamycin by knocking down Gal-1 expression [163]. This particular improvement of sensitivity appears to be regulated through P-glycoprotein expression via inhibiting the Raf-1/AP-1 pathway [163]. 

In triple-negative breast cancer (TNBC) cell lines, it was shown that Gal-1 was associated with doxorubicin sensitivity [164]. Doxorubicin is the first-line therapeutic in anti-breast cancer treatment, but toxicity and resistance remain an important concern clinically [166,167,168]. By silencing Gal-1, TNBC had decreased cell proliferation, migration, invasion, and doxorubicin resistance. This resistance was mediated through integrin β1/FAK/c-Src/ERK/STAT3/surviving pathway [164]. This implies that targeting Gal-1 in TNCB has great therapeutic potential and is likely to sensitize TNCB to doxorubin treatment at the same time. This is of importance as TNBC is particularly difficult to treat since it does not, or at low levels, express estrogen, progesterone, or Her2/Neu receptors—signaling pathways for many current USA Federal Drug Administration (FDA)-approved breast cancer therapeutics target. Overall this suggests that Gal-1 is a viable target to employ in MDR and TNBC, to be used for as a targeting strategy to deliver current conventional chemotherapeutics or via developing novel Gal-1 therapeutics.

In HCC, cells with genetically altered high expression of Gal-1 were more resistant to sorafenib as compared with isogenic low expressing Gal-1 cells, shown by knock-in as well as knock-out systems [91]. Sorafenib is a small molecule inhibitor of multiple tyrosine kinases, i.e., VEGFR, platelet derived growth factor receptor (PDGFR), C-and B-Raf FDA approved for the treatment of renal cell carcinoma and HCC. Survival curves for patients with low and high Gal-1 expression also correlated with sorafenib sensitivity and resistance, respectively.

Thus, these studies imply that targeting Gal-1 can be a powerful tool in combinatorial treatment strategies, overcoming single chemotherapy resistance, as well as in MDR cancers.

### 5.3. Radiation Resistance

Aside from Gal-1 overexpression in most cancers, this galectin is also upregulated by radiation [165,169,170] and hypoxia [171,172]. General hypoxia and local areas of hypoxia (<10 mmHg pO_2_) are prevalent in solid tumors and are negatively associated with cancer therapy success, compromising radiotherapy, and driving malignant progression [173,174]. Radiation requires oxygen to induce DNA damage through the generation of hydroxyl radicals and H_2_O_2_ [175] and hypoxic conditions can require up to three times the amount of radiation to generate the same effects (oxygen enhancement ratio) [176]. Low oxygen tension leads to stabilization of the protein transcriptional regulating, hypoxia inducible factor (HIF-1α), inducing downstream signaling and protein generation [96,171].

Since Chen et al. reported that H-Ras and HIF-1 interact [177], Kuo et al. [165] suggested a possible HIF1/Gal-1-positive feedback loop where HIF1 signaling under hypoxia can enhance Gal-1, which acts through H-ras to further promote HIF1 transcriptional activity [177,178]. Thus, they hypothesized that tumors may utilize this positive feedback loop to maintain elevated Gal-1 expression and HIF1 signaling to drive radio-resistance and aggressive tumor phenotypes [165].

Another way in which Gal-1 promotes radio-resistance is through its effect on the immune system, particularly on T lymphocytes. Gal-1 induces apoptosis in activated T lymphocytes through a CD45-associated *N*-glycan dependent manner [69]. Conversely, radiation enhances the antitumor immune response by increasing the generation of tumor-specific peptide or antigen repertoire and recruitment of cytotoxic T lymphocytes into the tumor [179,180]. However, radiotherapy does not always result in complete protective immunity, as relapse or recurrence is still a major clinical concern. Gal-1 potently induces apoptosis in activated T-cell resulting in an overall Th2 cytokine profile (e.g., Interleukin (IL)-4, -5, and -13) over a tumoricidal Th1 cytokine profile (e.g., INFγ, IL-2 and TGF-β) blocking immune effector functions while promoting IL-10-producing regulatory T cells to create an immune privileged site at the tumor [139,181]. Thus, the radiation-induced increase of Gal-1 levels promotes tumor immune evasion [165], limiting therapeutic response arguing combinatory treatment strategies of gal-1 inhibitors and radiation.

Indeed, Gal-1-induced radiation resistance has been overcome by using the Gal-1-targeting, designed peptide inhibitor anginex in multiple human and syngeneic murine tumor models [108,136,137,138,169]. Anginex synergizes with radiation, e.g., in the aggressive and radiation-resistant murine mammary carcinoma cell (SCK) model [138,156], the head and neck squamous cell carcinoma (SCCVII) model [147], the B16F10 melanoma model [144,156], and in the human ovarian cancer xenograft MA148 [138,144,156]. Moreover, the synergy of Gal-1 inhibition was observed with multiple radiation modalities. Namely, fractionated relatively lower dose radiation, i.e., multiple doses of e.g., 2 Gy, as well as when combined in a hypo-fractionated approach, e.g., a single or few doses of >10 Gy [138]. Moreover, using a radiation microbeam approach, it was noted that anginex sensitized tumors preferentially when combined with wider beam spacing radiation. Beam geometries and doses capable of slowing tumor growth were also more effective when combined with anginex [148]. In addition, multiple myeloma growth and its relapse can be repressed by using the anti-angiogenic agent anginex in combination with radiotherapy [182].

Thus, the radiation resistance induced by Gal-1 can be overcome by combining radiation therapy with Gal-1 inhibitors and shows great promise also in the contemporary radiation approaches such as hypo-fractionated and microbeam radiation. In addition, the combination of chemotherapy with temozolomide and radiation therapy has been reported to induce the expression of both Gal-1 and -3 [183] (Bailey et al., 2015). Thus, targeting both Gal-3 and -1 could have therapeutic benefit. 

## 6. Conclusions

Galectins are involved in many biological processes, generally functioning by interacting with various cell surface glycoconjugates, usually targeting β-galactoside epitopes. These small protein effector molecules mediate processes such as chemotaxis and angiogenesis, and thus have impact in various pathological disorders from cardiovascular disease to cancer. Whereas β-galactoside-directed glycan binding of galectins to various cell surface glycoconjugates is crucial to their extracellular biological functions, galectins are now also being recognized to mediate various intracellular functions via interactions with non-glycosylated nuclear and cytosolic biomolecules. Moreover, because their importance in biology has been growing rapidly in recent years, numerous efforts have been underway to identify effective antagonists of their function for use in the clinical setting. However only recently have galectins been fully accepted as valid therapeutic targets for clinical intervention. Even though this chapter has discussed a number of these drug discovery efforts, it is by no means exhaustive. It is likely that sometime soon, we will have one or more galectin antagonists available in the clinic to combat inflammatory diseases and cancer.

Note: Given the breadth of drug discovery and the galectin field of research with numerous labs involved in it, we apologize for the inadvertent omission of many excellent works.
